# Extending Body Space in Immersive Virtual Reality: A Very Long Arm Illusion

**DOI:** 10.1371/journal.pone.0040867

**Published:** 2012-07-19

**Authors:** Konstantina Kilteni, Jean-Marie Normand, Maria V. Sanchez-Vives, Mel Slater

**Affiliations:** 1 EVENT Lab, Facultat de Psicologia, Universitat de Barcelona, Barcelona, Spain; 2 IDIBAPS (Institut de Investigacions Biomèdiques August Pi i Sunyer), Barcelona, Spain; 3 Institució Catalana Recerca i Estudis Avançats (ICREA), Barcelona, Spain; 4 Department of Computer Science, University College London, London, United Kingdom; Royal Holloway, University of London, United Kingdom

## Abstract

Recent studies have shown that a fake body part can be incorporated into human body representation through synchronous multisensory stimulation on the fake and corresponding real body part – the most famous example being the Rubber Hand Illusion. However, the extent to which gross asymmetries in the fake body can be assimilated remains unknown. Participants experienced, through a head-tracked stereo head-mounted display a virtual body coincident with their real body. There were 5 conditions in a between-groups experiment, with 10 participants per condition. In all conditions there was visuo-motor congruence between the real and virtual dominant arm. In an Incongruent condition (*I*), where the virtual arm length was equal to the real length, there was visuo-tactile incongruence. In four Congruent conditions there was visuo-tactile congruence, but the virtual arm lengths were either equal to (*C1*), double (*C2*), triple (*C3*) or quadruple (*C4*) the real ones. Questionnaire scores and defensive withdrawal movements in response to a threat showed that the overall level of ownership was high in both *C1* and *I*, and there was no significant difference between these conditions. Additionally, participants experienced ownership over the virtual arm up to three times the length of the real one, and less strongly at four times the length. The illusion did decline, however, with the length of the virtual arm. In the *C2–C4* conditions although a measure of proprioceptive drift positively correlated with virtual arm length, there was no correlation between the drift and ownership of the virtual arm, suggesting different underlying mechanisms between ownership and drift. Overall, these findings extend and enrich previous results that multisensory and sensorimotor information can reconstruct our perception of the body shape, size and symmetry even when this is not consistent with normal body proportions.

## Introduction

Body ownership refers to the attribution of objects (e.g. limbs) as being part of one’s own body [1], [2], [3]. For example, for almost all people there is little doubt that their own arms or legs are part of their own body. However, there are neurological conditions where this seemingly basic property of self-attribution of the limbs breaks down, for example as in somatoparaphrenia [4], where there is the delusion that a limb belongs to another person, and other related disorders of the body scheme [5]. In this paper we show that it is possible to quite dramatically alter body representation, by inducing an ownership illusion over one very long arm in a situation where the entire body has been replaced by a virtual body seen from a first person perspective position (1PP).

It has been proposed that body ownership results from combining two types of knowledge: *prior* information that is innate or gained from the life experience (e.g. the appearance of our own body), and *current* multisensory information [6]. The motivation for such an approach has been provided by many studies showing that artificial objects can be perceptually incorporated as our own body parts when there are specific multimodal and/or sensorimotor events. For example, in the rubber hand illusion (RHI), synchronous visual stimulation of a rubber hand (placed in an anatomically plausible position on a table) and corresponding tactile stimulation of the hidden real hand induces the illusory feeling the rubber hand is part of the body representation [7], [8]. Under this illusory perception, participants when asked to localize their real hand they are more likely to point closer to the rubber hand after the stimulation compared to before [7], [9]. The difference between the participants’ estimations before and after the stimulation is called ‘proprioceptive drift’ and it is widely considered as a behavioral correlate of the ownership illusion, although a recent study [10] challenged the idea that there is a common underlying mechanism that connects drift with the subjective illusion of ownership. Similar illusory perceptions have been reported towards a live image of the real hand projected onto a table in front of participants [11], or a virtual hand that either receives the same stimulation as the real hand [12] or moves synchronously with it [13] - a virtual hand illusion (VHI). Generally the induction of ownership illusions requires multisensory stimulation with the same spatiotemporal pattern on the real and fake body part [7], [14]. Furthermore, it has been proposed that the artificial body part should obey various morphological, anatomical and postural constraints (for a review see [6]) including the necessity for human body part resemblance.

While these studies provide evidence that artificial objects that could plausibly be part of the body can be perceptually incorporated into the body representation, other studies show that neither the perceived body size nor shape is as rigid as we may believe. The illusion of having a body part e.g. the head, nose, chin, finger or waist as elongated or shortened can be induced when there is contact between, for example, the hand and this other body part while the subject has the illusion that the hand is extending away from or moving towards the body. The illusion of movement, induced by mechanical vibration of the biceps or triceps with eyes closed, requires the brain to resolve the contradiction between the moving end-effector and the contact with a non-moving body part such as the nose. It resolves this contradiction through the illusion of the body part (e.g., nose) becoming longer [15], [16], [17]. Also simultaneous vibration on the biceps and triceps muscle tendons can induce the perception of a shrunken arm [18]. However, the illusion of having a very long nose can be produced without such kinesthetic illusions. This occurs when a finger of a blindfold subject (S0) is manipulated by the experimenter to tap the nose of another subject (S1) who is sitting in front facing away from S0 while the experimenter simultaneously taps the nose of S0 [19]. Moreover, a recent study showed that it is possible to induce the illusion of having a very small or giant body using synchronous visuo-tacile stimulation on the visible dummy body seen from first person perspective and the unseen real body [20]. Similar methods were used to give normal sized men the illusion of ownership over a very fat virtual body, again seen from a first person perspective and with synchronous visuo-tactile and visuo-motor stimulation [21].

The same principle of synchronous multisensory stimulation has been used to address the question of whether we can assimilate asymmetries in the size of our normally perceived symmetrical body parts, for example our limbs. In [8] the RHI was induced using a fake hand 91 cm beyond the real one, although with a lesser intensity compared to when using a normal sized fake arm. In this study, it was found that the participants were aroused, as measured through skin conductance, when the distant fake hand’s finger was bent into a harmful position. Additionally it has been shown that it is possible to generate an illusion of ownership of a rubber hand that was 3 cm larger than the real hand [22]. Furthermore, it has been found that just seeing an artificial limb 20 cm longer and connected to the body could result in topographic reorganization of the primary somatosensory cortex [23].

In this paper we address the question of the extent to which an asymmetrical virtual body can be experienced as one’s own. To this end we induced a variant of the VHI on the dominant arm in five experimental conditions. In two of the conditions the dominant virtual arm was of the same length as the real one, and although the visuo-motor feedback was congruent in both conditions, in one the visuo-tactile feedback was congruent but not in the other. In the other three conditions the visuo-motor and visuo-tactile feedback were always congruent but the virtual arm was substantially elongated compared to the real arm. All conditions used immersive virtual reality where the participants also had a complete virtual body seen from 1PP. The elongation of the virtual arm involved strong asymmetry of the body, since the other virtual arm was at normal length. We show that a virtual arm of the same length as the real one is incorporated under congruent visuo-motor correlations but regardless of incongruence in the visuo-tactile information. Moreover, when all of the provided multisensory and sensorimotor input is congruent, a virtual arm up to three times the length of the real arm can be felt as part of the body representation, with a consequent alteration in the proprioceptive estimation of the hand position, and a defensive withdrawal movement evoked by a threat to the virtual arm near the distant hand position. Moreover, the evidence suggests that there is still about a 50–50 chance of an arm up to four times the real length being subjectively incorporated, but other evidence suggests that the illusion starts to break down at this length.

## Materials and Methods

### Ethics Statement

The experiment was approved by the Comissió Bioètica of the University of Barcelona, and all participants gave their written informed consent. The study was performed according to institutional ethics and national standards for the protection of human participants.

### Materials

**Figure 1:**
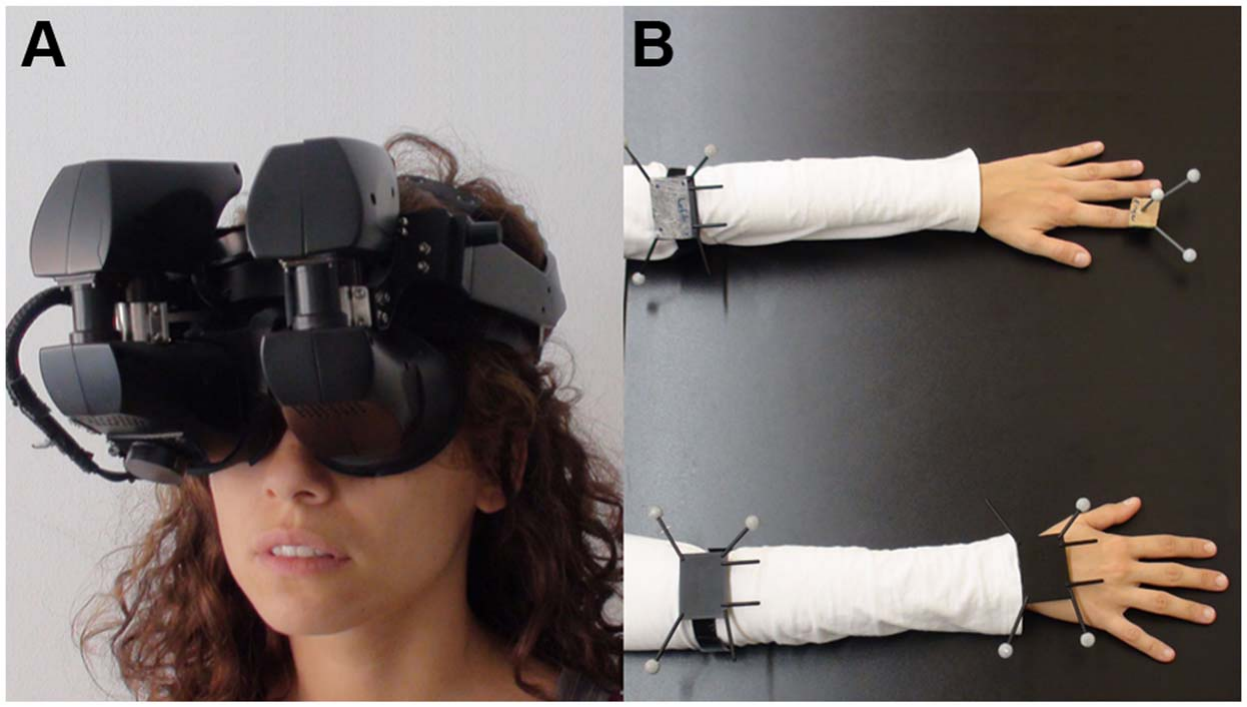
The Head-Mounted Display and Tracking. (A) Participants experienced the virtual environment through a stereo wide field-of-view Head Mounted Display. (B) Upper limbs were tracked by 12 Optitrack markers grouped in 4 trackable objects. The right and left forearms were tracked for all participants. For right handed people, the right hand and the left index finger were also tracked. For left handed people, the positions of the markers were swapped and thus the right finger and the left hand were tracked.

Participants were fitted with a stereo NVIS nVisor SX111 head-mounted display (HMD) (Figure 1A). This has dual SXGA displays with 76°H×64°V degrees field of view (FOV) per eye, totalling a wide field-of-view of 111° horizontal and 60° vertical, with a resolution of 1280×1024 per eye displayed at 60 Hz. Head tracking was performed by a 6-DOF Intersense IS-900 device. The dominant hand and forearm and the non dominant forearm and index finger were tracked with 12 infrared Optitrack cameras, which operate at sub-millimetre precision (Figure 1B). Full details of equipment can be found in Table S1.

**Figure 2:**
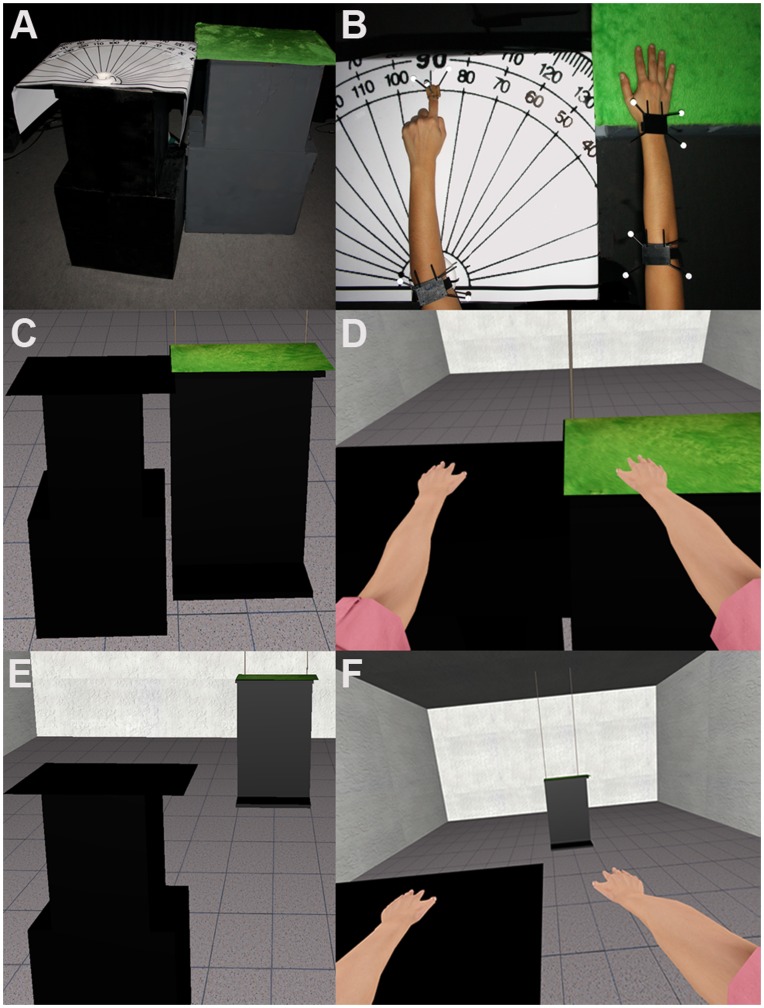
Spatial configuration of the physical and virtual scene. (A) There were two physical boxes, the Stimulus Box shown on the right and Angle Box shown on the left. For left handed people the positions of the boxes were swapped. (B) A plastic ring was attached on top of the Angle Box. The participant was asked to put his or her dominant hand on the Stimulus Box and the other one on the Angle Box with the elbow in the plastic ring. (C) There were virtual replicas of the physical boxes. (D) In all Congruent conditions, the virtual dominant hand of the participant was seen to touch the virtual Stimulus Box corresponding to the real hand touching the real Stimulus Box. In these conditions, when the participant moved the hand over the surface of the Stimulus Box feeling its material, the same movement was made and the same tactile feedback was seen. (E) In the Incongruent condition, the virtual Stimulus Box was placed 4 meters frontwards. (F) Therefore, although the virtual movement was the same as the physical, the virtual hand was never seen to touch the virtual replica of the Stimulus Box.

Participants were required to stand in front of two physical carton boxes that they never saw in reality. These were each (L×W×H) 70×50×116 cm^3^. One was covered by a light green felt (*Stimulus Box*) while on top of the other one a paper protractor to measure angles was placed together with an attached plastic donut-shaped ring used to keep the participant’s non-dominant elbow motionless on top (*Angle Box*) (Figure 2A, B).

All virtual models including a room, the two boxes, a saw, and male and female virtual bodies were modelled in 3D Studio Max 2010. The virtual environment was implemented on the XVR platform [24] and the virtual body was displayed using a hardware accelerated avatar library (HALCA) [25]. Inverse kinematics were used to ensure that when the participants moved their upper body the virtual body would move correspondingly. Spine and head rotations were calculated from the tracked head location and orientation. Forearm and upper arm rotations were calculated from the tracked hand position.

### Experimental Design

Fifty participants were recruited for the experiment by advertisement around the University campus. All participants answered a questionnaire giving demographic information before the experiment (see Table 1). In this questionnaire they were asked “Have you ever experienced ‘virtual reality’ before?” with possible responses on a scale from 1 (no experience) to 7 (extensive experience). Only 3 participants scored 4 or more, 5 participants scored 3, and 42 scored 1 or 2. All were compensated with 5€ after the end of the experiment.

**Table 1:** The Experiment Conditions.

Group	Visuo-tactile Correlation	Final Virtual arm length	Mean Age (mean ± SD)	No. right-handed	No. of males in group
I	Incongruent	1	23±3	8	4
C1	Congruent	1	22±5	6	2
C2	Congruent	2	22±4	8	2
C3	Congruent	3	22±4	7	3
C4	Congruent	4	22±4	7	3

n = 10 for each condition in a between-groups design. Congruent visuo-tactile correlation refers to the virtual arm being in contact with the Stimulus Box while the participant was touching it, and Incongruent refers to the virtual arm not reaching the Stimulus Box. In each condition there was visuo-motor synchrony between the real and virtual dominant arm.

The experiment included one factor and one independent variable. The factor was concerned with visuo-tactile correlations and had two levels: Incongruent (*I*) and Congruent (*C*). The independent variable, elongation, could take one of four possible values 1, 2, 3 and 4, corresponding to the ratio of the virtual arm length to the real arm length. We label these as *C1*, *C2*, *C3* and *C4* to make it clear that these were always in the Congruent condition. Hence *C1* refers to the virtual arm length being equal to the real arm length, and *C4* to the virtual arm being quadruple the real arm length. The condition *I* was only carried out with real arm length equal to the virtual arm length. Hence there were two conditions where the virtual arm was always the same length as the real one (*C1*, *I*). This was a between-groups design with 5 conditions as shown in Table 1. These five conditions can be conceptually considered as two different experiments. The first compared groups *C1* and *I,* thus testing the effect of congruence versus incongruence on the ownership illusion, everything else being the same. This was therefore a single factor with 2 levels between-groups design. The second experiment was to examine the effects of elongation on the virtual arm ownership illusion. Since this was a between-groups experiment group *C1* appears in both conceptual experiments. To be clear this group (and all others) only did the experiment once.

### Procedures

Before the experiment started, the experimenter measured the participant’s height and arm length. These values were used to set the virtual body’s height and arm length and served as the configuration for inverse kinematics. Participants wore the Head Mounted Display (HMD) and 12 Optitrack markers. The HMD was calibrated for each participant. The position of all participants was controlled and Velcro strips on the floor were used to mark where the participants’ feet should be located at the start of the experiment. These positions corresponded to the centre of the physical and virtual room. Participants were instructed not to move their feet or to move away from this position.

In this setup the body of the participant was represented by a gender-matched virtual body, of the same height as the person and with the same arm length. They saw this body only from 1PP, and therefore they never saw the head or face. Hence, when looking down they saw the virtual body as substituting their real one. For right-handed people, the Stimulus Box was positioned on the right of the participant at 50 cm depth and the Angle Box on the left at 20 cm depth and the opposite arrangement was used for left-handed people.

In all experimental conditions the virtual replicas of the boxes were of the same size as the real ones. In all Congruent conditions these were placed in the same initial position as the real box relative to each participant (Figure 2D). In the one Incongruent condition the virtual replica of the Stimulus box was placed at a distance of 4 m in front of the participant and therefore in a non-reachable position (Figure 2E, F).

Each participant was instructed to put his or her dominant hand on the Stimulus Box and the non-dominant elbow on the plastic ring of the Angle Box, aligning the forearm and hand to point forward (Figure 2B). They were instructed not to move their non-dominant arm but leave it motionless on the Angle Box in a fixed position with the elbow restricted inside the plastic ring. The motionless non-dominant arm and hand were co-located with the corresponding virtual arm and hand. The dominant virtual arm was also collocated with the real arm, and based on the tracking it also moved synchronously with the movements of the real arm.

The participant’s first task was to look around the virtual room in all directions, and in particular downwards to become aware of the full virtual body including legs and feet. During this visual exploration they were asked to state what they were seeing. They were then asked to describe the texture of the green material on top of the Stimulus Box by touching it. In order to do this they moved their real arm, but of course only saw the corresponding virtual arm move. In all the Congruent conditions the setup was calibrated such that they felt the texture of the surface on the box at the same time as they saw the virtual hand touch and move across the surface of the virtual box. Hence, all Congruent conditions provided synchronous visuo-motor and visuo-tactile correlations. In the Incongruent condition although the movement of the virtual hand and arm corresponded to the participant’s movements, the participant felt the box but saw that the virtual hand was not actually touching the virtual box. Hence the Incongruent condition provided synchronous visuo-motor feedback but induced a visuo-tactile mismatch (Figure 2F).

**Figure 3:**
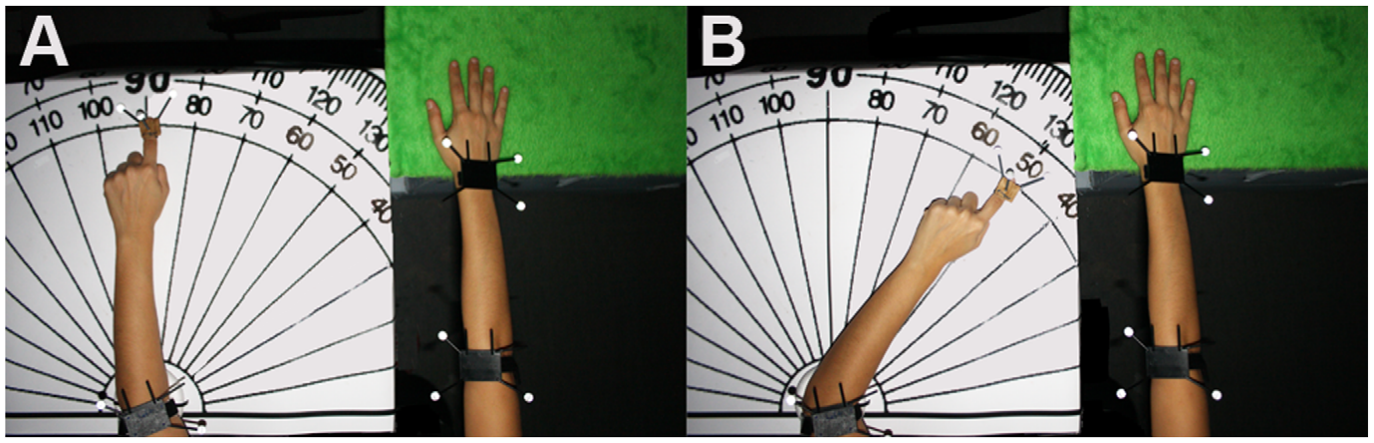
Estimation of the position of dominant hand using the non dominant arm. (A) The two limbs were aligned as shown pointing forward as shown, in the case shown here the dominant hand was the right hand. (B) The participant was instructed to rotate the non dominant arm to point with the index finger towards where they felt the other hand to be. The position of the elbow was restricted by the plastic ring. The angle was recorded. Each participant repeated this 10 times before elongation (to give the mean *AngleBefore*) and once after the elongation (to give the *AngleAfter*) for conditions *C2*, *C3* and *C4*.

In the conditions where the virtual arm elongated (*C2*, *C3* and *C4*) after the exploration phase the participants were asked to leave the dominant hand motionless on the Stimulus box, and as close as possible to the edge with the palm always touching the surface aligning the forearm-hand axis to the depth axis (Figure 3A). Participants had already been trained before the experiment started that whenever they were told ‘Please give me the angle’ the displays would become black and the they had to rotate the non-dominant elbow (restricted by the ring) and point towards the centre of the dominant hand (Figure 3B). The magnitude of the angle was recorded using both the tracking device and manually with the protractor. Immediately after that, the experimenter passively returned the participant’s non-dominant hand to the initial position and the display was switched on again. This procedure was repeated 10 times. For the two conditions (*C1* and *I*) where the virtual arm length was equal to the real arm length, these measurements were not taken.

**Figure 4:**
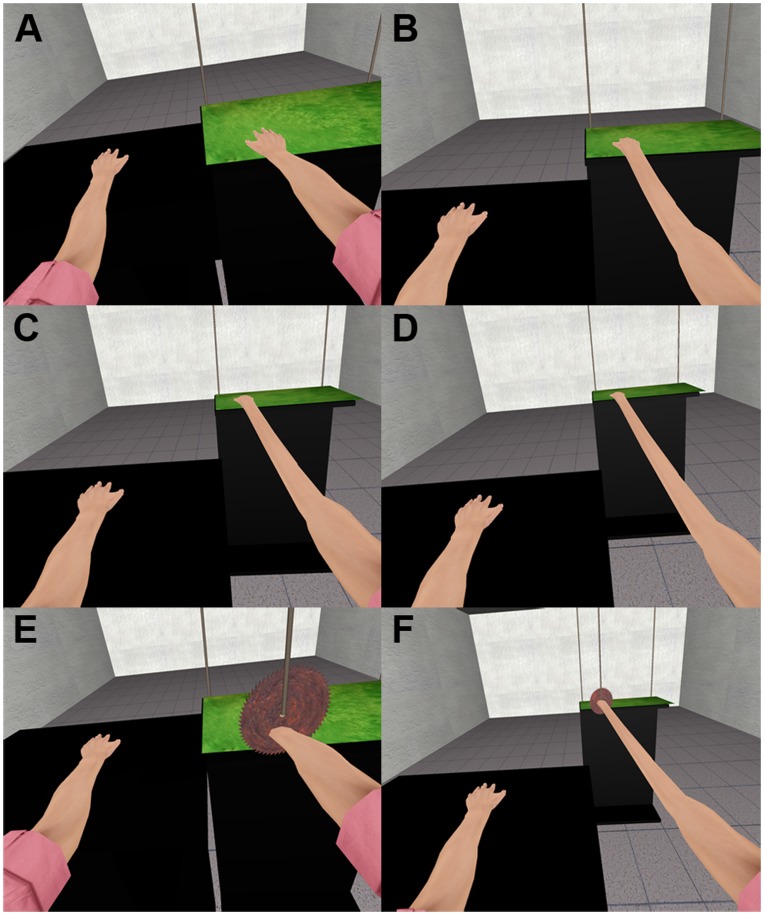
The elongation of the virtual arm and the threat event to the virtual hand. (A) At the start of the experiment, both virtual arms were of the same size as the participant’s arms. In *C1* the virtual arm did not change length during the experiment. (B) The arm elongated to double the true length (*C2*) (C) The arm elongated to triple the true length (*C3*) (D) The arm elongated to four times the true length (*C4*). When the elongation was complete for the condition and after the last angular estimation was made, a virtual saw fell to cut the virtual arm. The participants had been instructed to stay motionless just before this. (E) The position of the virtual threat was also close to the physical body and the real hand in the no elongation condition *C1* (F) The threat was far from the real body and real hand in condition *C4*.

The participant was then asked to continuously stroke the surface of the Stimulus Box with his dominant hand while still keeping his non-dominant arm motionless. In conditions *C2*, *C3* and *C4* the Stimulus Box moved away along the depth axis while the virtual limb correspondingly elongated to maintain the contact of the virtual hand on the top virtual Stimulus Box (Figure 4). The elongation step of the arm and the translation step of the Stimulus box were equal so that the virtual hand was always seen to be touching the virtual Stimulus box when the physical hand was touching the Stimulus Box thus maintaining visuo-tactile correlations. During the elongation period, participants were instructed eight times (every 15 seconds) to glance towards their non-dominant virtual arm, which served as a point of reference for the normal arm length. The elongation lasted two minutes to reach a final length equal to two, three or four times the arm length of the participant. The virtual arm length was always proportional to the arm length of each participant. Over all participants, the mean arm length was 53±3 (S.D.) cm with the elongation in *C2* therefore resulting in an arm of 106 cm, *C3* an arm of 159 cm and *C4* an arm of 212 cm, on the average. In those conditions where the virtual arm length did not change, the participant was therefore touching the real Stimulus Box for two minutes, with either Congruent (*C1*) or Incongruent visuo-tactile feedback (*I*).

After the 2 minutes of stimulation the participants in conditions *C2*, *C3* and *C4* only were again instructed to make one angular estimation using the same method as previously. In all conditions, participants continued touching the Stimulus Box with their dominant hand but were asked to leave it motionless and relaxed resting on the surface. After 15 s of this motionless period a rotating virtual saw appeared above the arm near the virtual hand position and dropped down towards the virtual arm and remained in a position as if cutting it for 4 s (Figure 4E, F).

After the virtual saw disappeared the participants were told “Now the experiment is finished” and for ethical reasons participants in the elongation conditions were shown the virtual limb at normal length for between 3 and 5 s and then the HMD was removed and participants were asked to complete a post-experimental questionnaire.

Each condition was recorded by video with written consent of the participants. For ethical reasons, two weeks after the experiment, all participants were contacted by email and asked about their experience in this experiment and whether they had any positive or negative thoughts about it. None of the participants experienced any negative post experimental sensations (Text S1).

An overview of the procedures can be seen in Video S1.

### Response Variables

There were three different types of response variable: a questionnaire, proprioceptive drift, and hand movement in response to the falling saw.

The questionnaire was based on that of Botvinick and Cohen [7] and available in English and Spanish. Participants were asked to rate 7 statements on a 1 to 5 scale where 1 indicated complete disagreement and 5 complete agreement with the statement. These were:

Q1. It seemed as if I were feeling the touch of the box in the location where I saw the virtual hand touching.

Q2. It seemed as though the touch I felt was from the box being touched by the virtual hand.

Q3. I felt as if the virtual arm were my arm.

Q4. It felt as if I might have more than two arms.

Q5. It seemed as if the touch I was feeling came from somewhere between my real and the virtual hand.

Q6. It felt as if my real arm were becoming longer.

Q7. I had the feeling that I might be harmed if the saw touched the virtual arm.

Q1 and Q2 relate to referral of touch to the virtual hand and Q3 is concerned with the subjective strength of the ownership illusion. Q4–Q5 were considered as the control questions and Q6–Q7 were considered as the questions referring to specific effects of the particular experiment. We expected, Q1–Q2 to be high in conditions *C1*–*C4* and significantly higher than in condition *I*. In our many (60) earlier pilots, we tested the induction of the illusion when elongating the arm by 1.5, 2, 2.5, 3, 3.5 and 4 times the real length. Ownership in these pilots was addressed only by a verbal report of whether they felt the illusion of having a very long limb and by any motor reaction in seeing the virtual saw cutting the virtual hand. We found that the illusion was weaker when the virtual arm was four times the real one. Hence, Q3, Q7 were anticipated to be high in conditions that could induce ownership namely in *C1*, *C2* and *C3* but lower in *C4*. Condition *I* involved aspects that could both support the illusion of ownership (visuo-motor correlations) and that could diminish the illusion (incongruent visuo-tactile correlations). Prior to the experiment we had expected that condition *I* would provide a lesser illusion of ownership than *C1* (and by implication *C2* to *C4*). We expected control questions Q4 and Q5 to be low in all conditions. Finally, Q6 was expected to be higher in the longer arm conditions (*C2*–*C4*) than in *C1* and *I*.

The measure of proprioceptive drift was based on the procedures that involved angle estimation as described earlier and was introduced to assess the effect of the virtual arm length on participants’ estimation, analogously to the effect of distance between the real and the rubber hand in the RHI. Since any difference between the pre and post stimulation estimation could be observed only in the presence of a discrepancy between the felt and seen position of the hand, e.g. when the arm length was different, this was added as an extra measurement only for the conditions *C2*, *C3* and *C4* (See Text S2). Our expectation was that the difference between the estimated position of the hand after the stimulation and the mean estimated hand position before the stimulation (angular drift) would correlate with the length of the elongation. *AngleBefore* denotes the mean and *SDAngleBefore* the standard deviation of the 10 angle estimates before the stimulation and can be considered as the ‘true’ angle pointing to the illuded hand. After the elongation participants were asked to indicate the angle again. *AngleAfter* denotes the single angular pointing direction following the manipulation. We expected the difference between *AngleAfter* and *AngleBefore* to be significant in *C2* and *C3* and less at *C4*, and also to correlate with virtual arm length. The magnitude of the angle was recorded using the tracking device and was also measured manually by the experimenter. Tracking data were used for all participants in conditions *C2*–*C4* except for 2 participants for whom the experimenter protractor based measurements were used due to technical failures in the tracking.

Response to the threat was based on tracking data collected around the period of the saw falling by recording the positions of the forearm and the hand. We distinguish between the Control Time a period of 2 seconds (120 samples) before the saw became visible and the Saw Time, 4 seconds (240 samples) while the virtual saw was actually in contact with the virtual arm. The time between the saw first appearing and when it touched the virtual arm is not useful for analysis because we have no way of knowing at which point participants actually noticed the knife entering their visual field. Therefore we rely on the Control and Saw Time periods.

In order to measure the amount of hand movement we use the square root of the mean squared distance between each point in the tracking data and the centroid, by analogy with the standard deviation of univariate data. Let
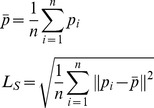
(1)where_
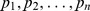
_ are the tracked points in the Saw Time and 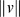 is the length of vector 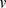. We similarly define 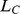 for the Control Time. We call these the hand tracking *dispersions*.

## Results

### Comparison of Congruent and Incongruent Conditions with No Elongation

Table 2 shows the medians and interquartile ranges of the questionnaire responses for the Congruent and Incongruent conditions when the virtual and real arm lengths are the same (*C1* and *I*). The referral of touch questions Q1 and Q2 result in significantly higher responses in the congruent compared to the incongruent condition, which would be expected according to the experimental design. However, there is no difference in median scores for the ownership question, and the median scores are both high (4 out of a maximum of 5). The only other response that shows a trend is Q7 (the feeling of being harmed) where the median is greater in the congruent condition (noting that the significance levels are for a two-tailed test). In this case the congruent score is very high (median of 4 out of a maximum of 5 with a low IQR).

**Table 2:** Comparison of Questionnaire Responses of the Congruent (*C1*) and Incongruent Conditions (*I*) with Virtual Arm Length Equal to Real Arm Length.

	*C1*		*I*		
Question	Median	IQR	Median	IQR	P
Q1	5	1	1	0	0.0009
Q2	4	3	1	0	0.0001
Q3	4	1	4	2	1.0000
Q4	1	0	1	0	0.6264
Q5	2	3	1	2	0.6177
Q6	2	2	1	1	0.1548
Q7	4	1	1.5	3	0.0914

P is the significance level for equal medians using a two-tailed Mann-Whitney U Test.

The recorded kinematic data were used to find any differences in motor activity in response to the threat of the saw. We compared the two periods, one as a control period before the saw was visible (Control Time) and then after the saw had reached the hand (Saw Time). Participants had been instructed to keep their hand still and relaxed throughout this period.

Table 3 gives the means and standard errors for 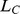 and 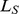 from which it is clear that there is no difference in the amount of hand movement between congruent and incongruent conditions. However, the mean Saw Time dispersion is greater than the mean Control Time dispersion for each group *I* and *C1* separately (bearing in mind that the significance levels are two-tailed) and for the two groups combined.

The evidence therefore suggests that the illusion of ownership was high for both the *C1* and *I* conditions. This is shown by the high median questionnaire score on Q3 and the responses to the falling saw.

**Table 3:** Means and Standard Errors of S (meters) for the Incongruent and Congruent Condition with Virtual Arm Length Equal to Real Arm Length.

Trial	Control Time *L_C_*	Saw Time *L_S_*	Ps-r
*I*	.0006045±.000427	.0013382±.000774	0.09
*C1*	.0005798±.000197	.0039828±.002920	0.09
Pr-s	0.17	0.54	
*Combined*	.0005921±.000229	.0026605±.001501	0.017

Ps-r is the two-tailed significance level for the Wilcoxon matched pairs sign-rank test.

Pr-s is the two-tailed significance level for the Wilcoxon rank-sum test. n = 10 participants in each cell, and n = 20 in the two combined cells.

### The Effect of Elongation

**Figure 5:**
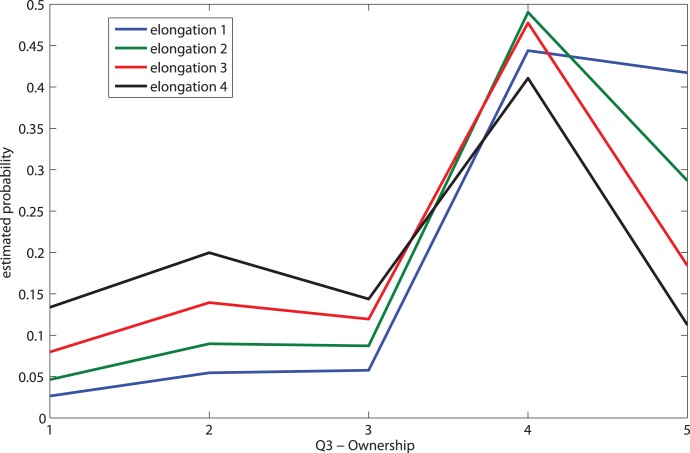
Estimated probabilities of the scores on the illusion of ownership (Q3). The probabilities are estimated from the fitted values of the ordered logistic regression of the Q3 scores on elongation.

We now consider only the conditions *C1*,…, *C4*– all the congruent conditions, but with the arm at the same length as the real one (*C1*), twice the length (*C2*), three times (*C3*) and four times the length (*C4*). First we show that the ownership question (Q3) is significantly associated with elongation. Table 4 shows the frequency table for ownership by elongation. Over the whole sample the level of reported ownership is high (28/40 have scores of at least 4 out of 5). For elongation 1 to 3 there are always at least 7 out of 10 participants with scores of at least 4. This declines to 4 out of 10 for elongation 4. We treat ‘ownership’ as an ordered categorical variable, and elongation as a numeric variable (since the values 1 through 4 refer to actual multiplicities of arm length) and carry out an ordered logistic regression of ownership on elongation (using Stata 12 software http://www.stata.com/stata12/). This results in a fit with negative coefficient (the greater the virtual arm length the lower the probability of being at a high level of ownership) with P = 0.032. The proportional odds assumption of logistic regression is not violated (using a Brant test [26], P = 0.59). Figure 5 shows the estimated probabilities from the logistic fit P(*ownership* = *i* | *elongation* = *j*), *i* = 1,…,5; *j* = 1,…,4. The estimated probabilities of the ownership score being 5 are 0.42, 0.29, 0.18 and 0.11 respectively for elongations 1 through 4. The estimated probabilities of the score being at least 4 are 0.86, 0.78, 0.66 and 0.52 respectively. It is clear that the probability is high for ownership to be scored at the highest level for equal length, but still at triple length the probability remains high, and only shows a decline at quadruple length. Nevertheless from Figure 5 the probability of a score of at least 4 at elongation 4 is estimated as about 50%.

**Table 4:** Frequency Table of Ownership by Elongation.

Q3	Elongation				
ownership	1	2	3	4	Total
*1*	0	1	2	0	3
*2*	1	0	0	4	5
*3*	1	0	1	2	4
*4*	5	5	4	4	18
*5*	3	4	3	0	10
*Total*	10	10	10	10	40

The mean and standard deviation of the pointing angle to the felt hand position sampled 10 times before the arm elongation, and the angle after the elongation were recorded as described in Methods at elongations C2–C4 to give *AngleDiff*  =  *AngleAfter* - *AngleBefore*. Although we did not measure angular drift in condition *C1* (no elongation but with congruence) we assume that the angular drift is 0 at elongation 1 - that is, when the arm is at the same length as the true arm there would be no angular displacement within random error. This can be justified by considering the data on the mean angle estimates for the 30 participants in conditions *C2*, *C3* and *C4*, each mean based on 10 trials. Figure S1 shows these means and their standard errors, and as can be seen the standard errors are very small compared to the means. The median of these 30 angle estimates is 45.6°, and the median of the corresponding 30 standard errors is 0.76° with interquartile range 0.59°. Hence the error in the pointing angle amongst the 10 estimates per participant is very small, which lends support to the assumption of 0 angular displacement. Then *AngleDiff* is positively associated with elongation (Spearman’s rank correlation is positive with P = 0.03, although the ordinary Pearson correlation is not significant, P<0.14 due to non-linearity, all significance levels two-sided).

**Figure 6:**
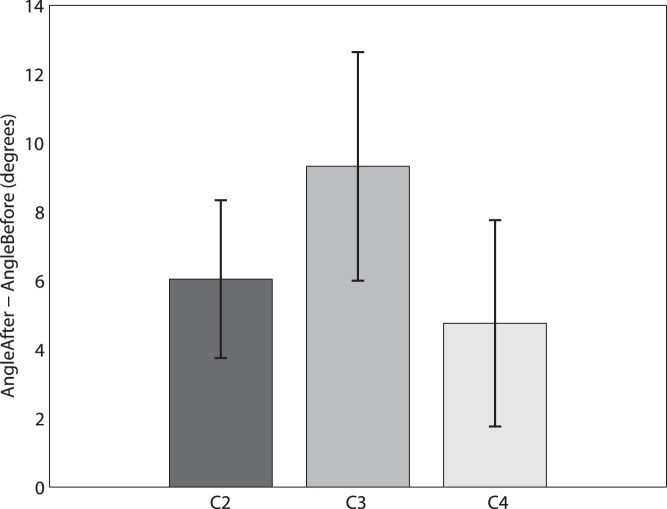
Means and Standard Errors of the Angular drifts for the elongation conditions. *AngleBefore* is the mean of 10 estimations of hand position at the start of the experiment. *AngleAfter* is the single estimation of hand position after the arm elongation period. *AngleAfter* is significantly greater than *AngleBefore* for *C2* (P = 0.04) and *C3* (P = 0.01) but not for *C4* (P = 0.17), Wilcoxon matched-pairs signed-rank tests.

Next we consider the angular drifts within each of the three elongation conditions *C2*, *C3* and *C4*. For each participant we take a conservative estimate of the upper bound of the prior hand position angle as the mean plus three times the standard error of the mean. Then for *C2*, *C3* and *C4* the number of participants with post-elongation angular drift greater than this were 7, 7 and 6 respectively out of 10. Figure 6 shows the mean angular displacements, all of which are positive. Using a Wilcoxon matched pairs sign rank test, we can test for the difference between the prior elongation angle estimate and the post elongation measured angle. The (two-sided) significance levels are for *C2* P = 0.037, for *C3* P = 0.012, and for *C4* P = 0.169. It should be noted that while the evidence supports the hypothesis that the angular drift was greater than the prior estimate for virtual arm length up to three times the true arm length, there is no correlation of the angular drift with any of the questionnaire responses.

**Figure 7:**
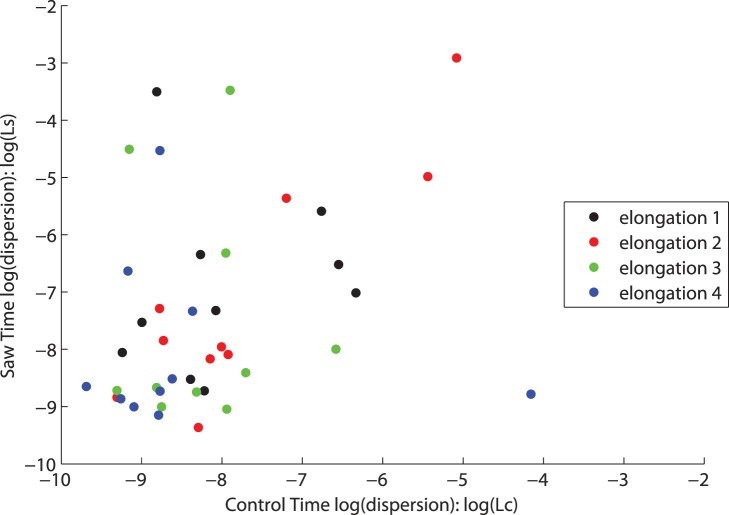
Scatter diagram of the Saw Time dispersion on the Control Time Dispersion by Elongation Condition on a log-log scale.

The dispersion of the hand positions as measured by tracking (Eq. 1) was greater during the Saw Time than in the Control time under all elongation conditions except *C4*, as shown in Table 5, and is significantly greater for *C1* and *C2*, and significantly greater for all elongation conditions combined. We now show that the dispersion in Saw Time declines with elongation, taking into account the dispersion during the Control Time. We would expect a relationship between the Control Time and Saw Time dispersions - due to propensities of participants to unwittingly make small movements, and simply due to noise in the tracking signal. Figure 7 shows the relationship between 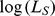 and 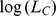 over the four elongation conditions (in the congruent condition). These are shown on a log scale to obtain approximate linearity. It can be seen that there are several outlying points. Also it can be seen that the relationship between the two variables may be different for different levels of elongation. For example, for equal and double length there appears to be a positive linear relationship (ignoring the outliers) but for triple and quadruple there may be no relationship. To investigate this further, as before we regress 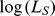 on 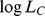 and *elongation* also including an interaction term 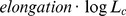 to allow for different relationships across the different lengths. Text S3 gives the results for normal regression, with detection and deletion of the outliers, showing a strong negative linear relationship between 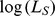 and *elongation* (taking into account the effect of 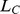). However, it did not prove possible to find a fit with normally distributed residual errors. A preferable way to approach this, without the necessity of identification and removal of outliers, is to use robust regression based on iteratively reweighted least squares [27]. The regression fit is shown in Table 6, supporting the notion of a negative relationship between the virtual arm length and the hand movement dispersion.

**Table 5:** Means and Standard Errors of the Hand Tracking Dispersions (meters) under the 4 elongation conditions.

Elongation	Control Time(*L_C_*)	Saw Time(*L_S_*)	P
*C1*	.000580±.000198	.00398±.00292	0.09
*C2*	.00129±.000681	.00681±.00533	0.07
*C3*	.000366±.000120	.00449±.00312	0.72
*C4*	.00169±.00156	.00138±.00105	0.20
*Combined*	.000984 |±.000420	.00417±.00169	0.01

P is the two-tailed significance level for the Wilcoxon matched pairs sign-rank test.

**Table 6:** Robust Regression for *E*(log *L_s_*)  =  *β*_0_ + *β*_1_ *elongation* + *β*_2_ log *L_c_* + *β*_3_ *elongation* log *L_c_*

Coefficient	Estimate	P (t-test)
*β*_0_	1.46	0.605
*β*_1_	−3.19	0.027
*β*_2_	1.09	0.004
*β*_3_	−0.36	0.041

This uses the robustfit function of MATLAB R2009a.

Further meaning can be attributed to the hand movement dispersion. It is positively related both to Q3 (ownership) and Q7 (harmed). A regression of 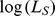 on 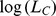, *ownership* and 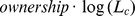 shows a positive slope for ownership (P = 0.0058) but no significant effects for the other two variables. However, the residual errors are not normally distributed (Shapiro-Wilks test, P = 0.00005). Dropping the two non-significant terms results in a significant positive slope for ownership (P<0.01), but does not resolve the problem of the non-normality of the residual errors. However, a robust regression of 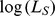 on *ownership* and 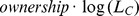 results in a highly significant positive slope for both variables (P<0.0005 in both cases). The case of *harmed* is more straightforward – there is a positive linear regression slope of 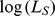 on *harmed* (P<0.0001) with the residual errors satisfying normality (Shapiro Wilks P = 0.272) with 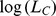 and 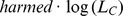 not significant.

It should be noted that these correlations do not extend to the remaining questionnaire variables.

### Putting It All Together

In the sections above we have used traditional linear model single equation techniques to investigate the relationship between a number of variables - the degree of elongation, the sensation of ownership, the feeling to be harmed, the amount of movement before and during the time of the saw on the hand, and the angular displacements. However, the relationships between these variables are likely to be far more complex than can be accounted for by single equation models. For example, the sensation of ownership may influence the degree to which participants had the feeling that they may be harmed, which in turn may influence the amount of hand movement, and the amount of hand movement may also be directly influenced by the feeling of ownership. The extent of elongation may influence the degree of ownership, and may directly influence the feeling of being harmed, and so on. Using standard linear models it is impossible to unravel such multiple interrelationships.

Instead we use path analysis to bring the various results reported above into one overall framework. For this purpose we treat all of the questionnaire variables as if on an interval scale. This is very typically done, and although not strictly justified, it provides a useful exploratory tool, and also the problem is lessened by the use of non-parametric statistics.

**Figure 8:**
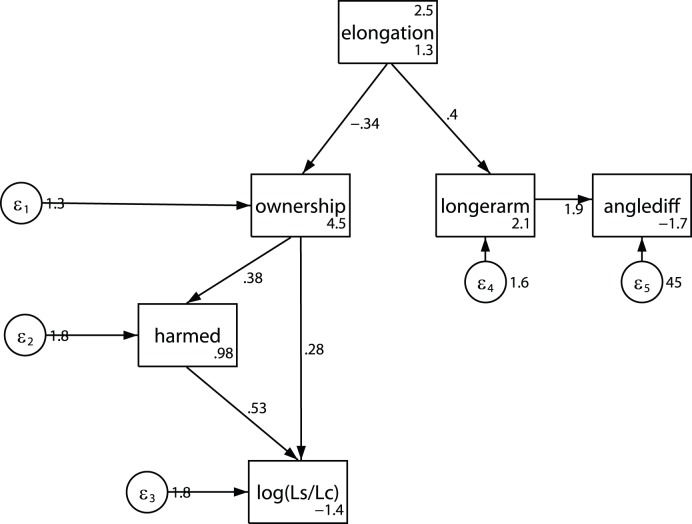
Path analysis diagram. The boxes represent the variables where *elongation* is 1 to 4, *ownership* is the response on Q3, *harmed* is the response on Q7, and 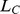 and 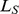 are the dispersions in Control Time and Saw Time. The variable *longerarm* is the response on Q6, and *anglediff* is *AngleBefore*-*AngleAfter*. The paths represent the regression lines, where, for example, 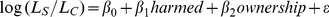 where 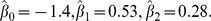 The circles represent the random error terms and the corresponding numbers are the variances of the errors.

Path analysis was carried out using the Structural Equation Modeling software of Stata 12. Estimates and significance levels were computed using the asymptotic distribution free option, which does not rely on underlying multivariate normal distributional assumptions. The path model is shown in Figure 8 with corresponding details of estimates in Table 7. At first we included all paths according to our prior expectations about relationships between the variables. Then those paths that were not significant were eliminated until only significant paths remained. A path from *elongation* to 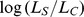 was originally included which showed a positive trend (P = 0.065) and from *elongation* to proprioceptive *drift* which was positive but not close to significance (P = 0.133). These were deleted because their P values were much greater than those of the remaining paths.

**Table 7:** Path Model Estimates Corresponding to Figure 8.

Variable	Coefficient	S.E.	P
***ownership:***			
*elongation*	−0.341	0.132	0.010
*intercept*	4.489	0.358	0.000
***harmed:***			
*ownership*	0.377	0.115	0.001
*intercept*	0.978	0.353	0.006
***log(L_S_/L_C_):***			
*ownership*	0.283	0.110	0.010
*harmed*	0.532	0.162	0.001
*intercept*	−1.424	0.472	0.003
***longer:***			
*elongation*	0.405	0.135	0.003
*intercept*	2.134	0.413	0.000
***anglediff:***			
*longerphysical*	1.888	0.596	0.002
*intercept*	−1.673	1.495	0.263

n = 40, Chi-squared goodness of fit  = 12.48, d.f.  = 9, P = 0.19.

The path model shows that an increase in the degree of elongation is associated with a decrease in ownership. However, at virtual arm length equal to the real one the fitted value of ownership is 4 and at quadruple the size the value is 3.3. Although there is a decline is it is not a great one, in line with the previous observation that when the virtual arm has the quadruple length there is still about a 50% chance of a high score for ownership.

Ownership is positively associated with harmed, which is in turn positively associated with the relative hand dispersion. Ownership is independently also directly positively associated with the relative hand dispersion.

Elongation influences greater proprioceptive angular drift but via the sensation that the real arm has grown longer (Q6). The questionnaire responses for Q6 are available under all experimental conditions, but recall that the angle drift measures are only available for *C2* to *C4*. Hence to be safe we could delete *anglediff* from the path model. The significance level from elongation to Q6 is of course still P = 0.003.

The path is in two separate parts, on the left relating elongation to ownership and the responses to the falling saw, and on the right hand side to the length of the arm and corresponding angle to the hand. Other than through elongation there is no other connection from the left side to the right side. For example, if a path from *ownership* to *longerarm* is added to the model, this path has significance level only at P = 0.212. Thus the path model supports the notion that the sensation of ownership and the angular proprioceptive drift are based on different underlying mechanisms.

## Discussion

The current study provides direct evidence that our body representation can be altered significantly through a pattern of multisensory and sensorimotor stimulation that is spatially and temporally consistent with such an altered body representation. Congruent multisensory and sensorimotor feedback between the unseen real and the seen virtual arm, can induce sensations that the seen arm is part of the actual body representation. More interestingly, the presence of the same correlations can induce the illusion even when the seen virtual limb triples in length, as reported by the participants’ responses in a questionnaire. Also, we found that proprioceptive drifts were affected by the length of the virtual arm and that defensive motor responses under a threat towards the virtual limb also adapted to this new body image, indicated by a defensive withdrawal movement in response to the falling saw.

In the first experiment we compared congruent with incongruent visuo-tactile feedback by including a condition where the real hand felt the box, but the virtual hand, although congruent in its movements, was not seen to touch the virtual box even while the real hand was touching it. Here, we extended the results of the VHI [12], [13] by showing that a virtual arm with same length and laterality as the real arm can become part of the body representation when it moves with the same spatiotemporal pattern as the real one and that under this condition it does not matter whether it receives congruent or incongruent visuo-tactile feedback. These sensations were reported in questionnaire results and observed through participants’ motor defensive reactions in response to a threat towards the virtual hand.

Before discussing the results of the first experiment, it would be useful to mention the differences between the setup used in *C1* and *I* and the typical setup in a RHI study. First of all, instead of a fake limb, a whole fake body seen from 1PP was displayed and explored before any stimulation. Participants saw a complete virtual body overlapping their real one and not just a virtual hand. However in both setups, the fake hand is seen connected to the rest of the body. Secondly, in the present experiment, apart from the visuo-tactile information (either congruent or not), active visuo-motor correlations were also provided and these were always congruent. Here, participants could move their arm and see congruent movements of the virtual one. This information is in favor of perceiving the limb as part of the actual body representation and it is additional information that is not provided in the classic RHI studies. Indeed, voluntary action has been proposed to cohere the sense of body ownership [2] and it has been demonstrated that efference facilitates self-recognition [28]. Third, the nature of visuo-tactile information given from the current design is different from the one provided in the RHI. While in the RHI the tactile stimulation is delivered by the experimenter, here the stimulation was self-generated. This, on one hand, might have augmented the predictability of the feedback since participants could have a certain expectancy about what they would see when touching the physical box through the efference copy. On the other hand, the possibility of making voluntary actions with congruent feedback should have induced the sense of agency and control towards the virtual limb. Finally, in the incongruent condition (*I*), the virtual limb was never seen to touch anything. Therefore, for all the above reasons, the incongruent condition (*I*) cannot be considered as equivalent to the asynchronous condition typically used in the RHI.

Condition *I* was designed initially to serve as a control condition. The fact that the illusory ownership sensations were induced even in this incongruent condition can have several possible explanations. First it might be thought that participants did not perceive the visuo-tactile incongruence. However, responses to Q1 and Q2 about touch referral were significantly different between the two conditions *C1* and *I* showing that the participants had perceived the mismatch between seen and felt touch in *I*. Secondly, it may be the case that when there is good correspondence in size and posture between the real and virtual body, and therefore also they are very ‘close’ to one another (essentially coincident) that all sensory correlations do not need to be completely in line with one another. This supports the findings of [29] that the closer together the surrogate and real arm, the less important is synchronous visual-tactile information in order to induce the illusory feeling of ownership. Another explanation could be that first person perspective together with active visuo-motor correlations provide a sufficient condition for an ownership illusion and overrides visuo-tactile inconsistencies. Considering the fact that participants in the Incongruent condition were touching the Stimulus box and moving their arm during the whole stimulus period, they therefore spent the same time experiencing 1PP, visuo-motor consistency and visuo-tactile incongruence. Hence, the effect could be due to the different relative importance of these factors. Such an account is in line with the findings in [30], [31] that first person perspective dominates visual-tactile synchrony in its contribution towards body ownership illusions. Such an interpretation is highly probable especially because the movements of the participants were self-generated (participants made active movements).

In three of the five trials participants were moving their dominant hand over the surface of the box, while the virtual box started to slide away with the virtual hand remaining in contact with the box and in synchrony with the movements of the real hand. The virtual arm elongated corresponding to the position of the box to eventually reach a length that was 2, 3 or 4 times the length of the real arm. Questionnaire scores for ownership were high in condition *C2* and *C3* and less in *C4*. Additionally, proprioceptive drifts and the difference in the dispersion of movement data were significant in *C2* and *C3* but not in *C4*.

Two further critical points should be considered: First, the elongation of the virtual arm could not have been perceived as a displacement of the entire visual field as if the participant had been looking through a prism and therefore distorting the whole scene along the depth axis [32]. The virtual non-dominant arm was always present in the visual field serving as a point of reference for the limb’s normal length and participants were instructed to look occasionally towards the non-dominant arm. In this manner, the asymmetry between the lateral limbs was emphasized. The rest of the virtual body was also visible. And indeed, all participants in the conditions with the long virtual arm perceived an elongation of the dominant virtual limb and not a distortion of visual space in the depth axis.

Second, although there were statistically significant hand movements as measured by the movement dispersion in response to the falling saw compared to the period before the saw fell, the magnitude of these defensive movements were small. It should be noted that participants had been instructed to be motionless in the final phase of the experiment. Thus, it is very probable that there was a competition between staying motionless and avoiding the saw. As reported by many of the participants, the instruction to be motionless inhibited the full execution of the defensive movement. Video evidence (see Video S1), suggests that moving the hand was an automatic withdrawal response. Our data indeed reflect motor initiation but probably not full execution of the defensive movement for some of the participants.

In trials *C2* and *C3* there were high scores for ownership and a threat far away from the physical hand but close to the virtual hand at the end of the very long arm that triggered the participants’ body defensive mechanisms. Regression analysis revealed that withdrawal movements were positively correlated with Q3 and Q7, validating the use of withdrawal movements to address ownership. Both measurements confirmed that an illusion of ownership was induced for an arm up to 3 times the length of the real arm. These findings revealed that the body representation is flexible and that it is possible to feel ownership towards a transformed and very asymmetric body that contradicts all the notions one has about the human body. This further extends the findings of [20], [21] that it is possible to generate the illusion of ownership of quite a different body size and shape compared to normal, except that here there was a strong virtual body asymmetry. Although it has been proposed that body ownership is governed by top-down mechanisms assuring that the human form is maintained [6], [9], we support the view that ownership can be considered to be determined at any moment of time as a relative balance between prior knowledge about human body form and current multisensory and sensorimotor information. The current results reveal that multisensory and sensorimotor input that gives evidence about limb size that diverges greatly from the normal limb size is sufficient to induce a body transformation illusion reported here by both perceptual and motor responses. Prior knowledge concerning the human limb size was obviously violated in this experiment, revealing that multisensory and sensorimotor stimuli drove this bodily illusion.

However if the body representation is so flexible, why was there some evidence of a diminishing ownership illusion when the virtual arm was 4 times the size of the real? In trial *C4*, decay in the intensity of illusory sensations was observed when the virtual arm was four times the length of the real arm, on average across the participants the length being 212 cm. There is evidence from the questionnaire supporting a decline in ownership at this length compared to the normal length. Additionally, dispersion of the movement data during the Saw Time did not differ significantly from the Control Time. One issue is that the virtual hand was very far from the rest of the body and hence its visual precision and quality were poorer compared to the other conditions, e.g. the longer the arm, the more difficult to see hand’s details. The hand was always seen to touch the virtual box even when it was four times the distance of the real one, but of course the closer the hand, the greater the visual information. Furthermore, it could be stated that the virtual hand is very far from the rest of the body, which might be also a limitation for the induction of the illusion. In [33] for example, there is an argument in favor of spatial limitations in the RHI since there was a decay in the illusion intensity together with an increase in its onset when the discrepancy in the position and the orientation of the two hands was increased. However, this hand was very far from the rest of the body also in *C2* and *C3*. Finally, it could be argued that there might be a limitation in the flexibility of the body limbs representation depending on the length. For example, in the study of [8] the intensity of the RHI was smaller when a distant fake arm was used (+91 cm) compared to a normal size fake arm as indicated by the questionnaire responses but not from skin conductance responses. In our study an ordered logistic regression of ownership (Q3) on elongation revealed a negative relationship between illusion and length, i.e. the longer the arm, the less probable ownership to be induced as shown in Figure 5, which is in line with the small decay found in *C4* and with the results of [8]. The same was also true for withdrawal movements and elongation. Accordingly, we support the view that the more extreme the body distortion the richer the sensory information that might be needed in order to induce the illusion of ownership. We therefore propose that a new experiment providing richer multisensory and sensorimotor stimulation the greater there is distortion from the true body shape, could establish the illusion for even greater lengths.

In line with the findings from questionnaires and hand movement data, proprioceptive drifts were found to be significant only for *C2* and *C3*. Although these revealed that participants overestimated the angle towards the position of their real hand, this was not found to be correlated with the illusion of ownership. A path analysis was carried out to investigate multiple overall dependencies. Proprioceptive drifts were found to be affected by elongation but only through the sensation that the real arm feels longer and not through ownership. This suggests that ownership and drift are based on different underlying mechanisms, as proposed also in [10]. In other words, overestimating the position of the hand does not necessarily imply the illusion of ownership of a longer arm. Although the experimental setup and the method of measurement used in [10] was different from here and the real hand was stationary, we consider that the present results strengthen the argument that there are probably different underlying mechanisms of drift and the ownership.

What are the theoretical implications of having a long arm? First, it would influence the perceived spatial configuration of the body since the brain integrates the available multisensory input taking into account the size of the body parts [34]. Secondly, neurophysiological studies on monkeys (for a review, see [34]) and behavioral studies in humans e.g. [35] have shown that the brain encodes personal space (i.e. the space occupied by our body) differently from the peripersonal space (i.e., the space adjacent to the body that is within arms’ reach), and also from far non-reachable extrapersonal space. Therefore, one would expect that perceiving limbs of different sizes would influence the perception of body space. Additionally, peripersonal space has been shown to be encoded in body-part reference frames [36], [37], [38], [39], [40] and is thought to serve as a safety zone for the body, adapted to the body shape [41]. Consequently limbs of different sizes would also redefine our margin of safety since accurate information about limb size is relevant to navigate through space while avoiding obstacles and harmful collisions. The triggering of the participants’ body defensive mechanisms when the virtual hand was under threat, implies firstly that the long arm was perceived indeed as a body part and not as a tool simply extending the peripersonal space [42]. Similar measurements for testing the feeling of embodiment in extracorporeal structures have been used in earlier studies [8], [30], [31], [43], [44]. In these studies, the artificial limb or body was threatened and the physiological or motor responses of the subjects were measured. From these studies however, it is not clear whether subjects respond because the threat is perceived *close* to their actual body space, which is normally similar in size and position to the illusory one. In the present study, the falling saw occurred far away from the physical body space varying on average from 53 cm (*C2*) to 159 cm (*C4*) from the real hand. In other words, participants tried to remove their hand automatically even when the harmful event occurred very far from their physical body space. These findings imply that the visual space was re-encoded in the virtual hand reference frames with a recalibration of the body segmental and postural configuration and a consequent update of the personal and peripersonal space under the perception of having a very long arm.

It should be noted that in the present study the arm grew continuously from its real length to the final length. There have been previous studies where there was an attempt to induce body size illusions where subjects were shown an already distorted body part from the outset, although the degree of distortion was not of the same magnitude as the one described in this paper. For example, the experiment in [8] showed a long fake arm from the outset of their experiment and the same was the case in [23] where a long fake arm was put just in front of the participants seen to be connected with their body. In [20], [21] an already distorted artificial body was shown from the outset. Our study differs from these in the sense that the virtual body was initially well proportioned and adapted to the participants’ real bodies but gradually and continuously grew in length. Here, during the elongation phase, each image of the virtual arm was different from the previous one inducing the sensations of having an arm that continuously extended but still moved synchronously with the real one and received congruent and synchronous tactile feedback in relation to a box that would not be reachable in reality. Additionally, it was not the whole body that was scaled up or down but one body limb elongated while the contralateral one maintained its length. Recently [45] induced an illusion of having a finger continuously stretched to double its size.

Rapid changes in the primary somatosensory cortex (SI) due to visual exposure to a long artificial arm were found in the study of [23] where just the visual impression of having an elongated arm resulted in modulations of the cortical hand representation in SI. More interestingly, this modulation was significantly and positively correlated with the magnitude of the subjects’ sensations of having a long arm. We speculate that such changes in activation of SI might have been also observed under the present experimental study. Future studies should address this issue testing the amount of cortical reorganization under various lengths of the virtual limbs.

## Supporting Information

Figure S1 Means and Standard Errors of the Angle estimation for each of the 30 participants in the elongation conditions *C2*, *C3*, *C4* before the elongation. Each value is based on 10 trials by each participant.
(EPS)

Table S1 Equipment Details.
(PDF)

Text S1 Post Experiment Ethics Check.
(PDF)

Text S2 Drift and Discrepancy in Real and Virtual Hand Conditions.
(PDF)

Text S3 Regression Analysis for Saw Time Dispersion log(LS).
(PDF)

Video S1 Highlights from the Experiment. This video illustrates the major parts of the experiment.
(MP4)
