# Supplementary material for: Extending Body Space in Immersive Virtual Reality: A Very Long Arm Illusion
Source: PLoS One. 2012 Jul 19;7(7):e40867. doi: 10.1371/journal.pone.0040867 (PMC3400672; doi:10.1371/journal.pone.0040867)
Supplement: Table S1 — Equipment Details. (PDF) [file pone.0040867.s002.pdf]

SUPPORTING INFORMATION

Supporting Table S1

Equipment Details

**Equipment**	**Company name**	**Website**
Head mounted display	**NVIS**	http://www.nvisinc.com/product.php?id=48
Head tracking	**INTERSENSE**	http://www.intersense.com/pages/20/14
Motion capture hardware	**Natural Point Optitrack**	http://www.naturalpoint.com/optitrack/products/v100-r2/ http://www.naturalpoint.com/optitrack/products/suits-markers/
Motion capture software	**Natural Point Optitrack**	http://www.naturalpoint.com/optitrack/products/tracking-tools/
Video capture	**Sony**	http://www.sony.es/product/hdd-avchd-hard-disk-drive/hdr-xr520ve
