# Supplementary material for: Extending Body Space in Immersive Virtual Reality: A Very Long Arm Illusion
Source: PLoS One. 2012 Jul 19;7(7):e40867. doi: 10.1371/journal.pone.0040867 (PMC3400672; doi:10.1371/journal.pone.0040867)
Supplement: Text S1 — Post Experiment Ethics Check. (PDF) [file pone.0040867.s003.pdf]

Supporting Text S1

Post Experiment Ethics Check

All participants were contacted 2-3 weeks after the experiment by email and they were asked to answer the following questions:

- Did you think about the experiment after having done it?
- Did you have any relevant feelings related to the experiment?
- Did you have any negative thoughts about the experiment?
- Did you have any positive thoughts about the experiment?
- Did you have any strange feeling, thought or behavior related to the experiment?

Nobody reported any negative or strange carry over effects due to the experiment. Most of them found the experiment very interesting and curious.
