# Supplementary material for: Extending Body Space in Immersive Virtual Reality: A Very Long Arm Illusion
Source: PLoS One. 2012 Jul 19;7(7):e40867. doi: 10.1371/journal.pone.0040867 (PMC3400672; doi:10.1371/journal.pone.0040867)
Supplement: Text S2 — Drift and Discrepancy in Real and Virtual Hand Conditions. (PDF) [file pone.0040867.s004.pdf]

Supporting Text S2

Drift and Discrepancy in Real and Virtual Hand Conditions

In a typical RHI setup, there is a discrepancy between the real and the rubber hand positions and the measured drift can appear because of the distance between the two hands. In situations where the rubber hand is projected to be at the same position of the real hand [1,2], there is no discrepancy in position and thus the estimated value of the participant should not vary before and after the stimulation, independently on whether the illusion was induced or not. Analogously in conditions *C1* and *I* there was no discrepancy in the position of the real and fake hand and thus, any measurements would be uninformative.

1. Zopf R, Savage G, Williams MA (2010) Crossmodal congruency measures of lateral distance effects on the rubber hand illusion. Neuropsychologia 48: 713-725.
2. Hohwy J, Paton B (2010) Explaining away the body: Experiences of supernaturally caused touch and touch on non-hand objects within the rubber hand illusion. PLoS ONE 5: e9416.
