# Supplementary material for: Extending Body Space in Immersive Virtual Reality: A Very Long Arm Illusion
Source: PLoS One. 2012 Jul 19;7(7):e40867. doi: 10.1371/journal.pone.0040867 (PMC3400672; doi:10.1371/journal.pone.0040867)
Supplement: Text S3 — Regression Analysis for Saw Time Dispersion log(LS). (PDF) [file pone.0040867.s005.pdf]

Supporting Text S3

Regression Analysis for Saw Time Dispersion $\log(L_S)$

Regression equation: (n = 40) for all the congruent conditions *C1*,…,*C4*

$$\log(L_S) = \beta_0 + \beta_1 elongation + \beta_2 \log L_c + \beta_3 \log L_c \cdot elongation + \varepsilon$$

The regression equation yields the following results, with $R^2 = 0.19$.

$\log(L_s)$	Coefficient	S.E.	P
$\hat{\beta}_0$	0.516	3.145	0.870
$\hat{\beta}_1$	-2.775	1.565	0.085
$\hat{\beta}_2$	0.928	0.393	0.024
$\hat{\beta}_3$	-0.305	0.190	0.117

This indicates a trend for elongation where greater elongation is associated with less dispersion in the Saw Time.

However, the Shapiro-Wilk test for normality of the residuals gives P = 0.00003, clearly rejecting the hypothesis of normality. The plot of residual errors against fitted values below clearly suggests 5 outliers, those points with residual error > 2.

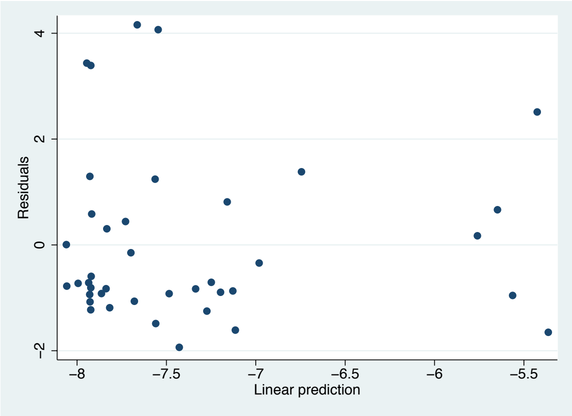

Residuals by Fitted Values

When the model is refitted with these outlying points removed the new fit is (n=35):

$\log(L_s)$	Coefficient	S.E.	P
$\hat{\beta}_0$	0.082	1.962	0.967
$\hat{\beta}_1$	-2.825	0.902	0.004
$\hat{\beta}_2$	0.930	0.246	0.001
$\hat{\beta}_3$	-0.307	0.110	0.009

with $R^2 = 0.44$. However, the Shapiro-Wilks test still rejects normality at P = 0.014. It is therefore preferable to use a robust regression, since no other transformation could be found that leads to normality of the residual errors.
